# Biomarkers of endothelial dysfunction, cardiovascular risk factors and atherosclerosis in rheumatoid arthritis

**DOI:** 10.1186/ar1717

**Published:** 2005-03-24

**Authors:** Patrick H Dessein, Barry I Joffe, Sham Singh

**Affiliations:** 1 Department of Rheumatology, Johannesburg Hospital and Milpark Hospital, Parktown, University of the Witwatersrand, Johannesburg, South Africa; 2 Centre for Diabetes and Endocrinology, Houghton, University of the Witwatersrand, Johannesburg, South Africa; 3 Lancet Laboratories, Richmond, Johannesburg, South Africa

## Abstract

Cardiovascular event rates are markedly increased in rheumatoid arthritis (RA), and RA atherogenesis remains poorly understood. The relative contributions of traditional and nontraditional risk factors to cardiovascular disease in RA await elucidation. The present study comprises three components. First, we compared biomarkers of endothelial dysfunction (vascular cell adhesion molecule [VCAM]-1, intercellular adhesion molecule [ICAM]-1 and endothelial leucocyte adhesion molecule [ELAM]-1) in 74 RA patients and 80 healthy control individuals before and after controlling for traditional and nontraditional cardiovascular risk factors, including high-sensitivity C-reactive protein (hs-CRP), IL-1, IL-6 and tumor necrosis factor-α. Second, we investigated the potential role of an extensive range of patient characteristics in endothelial dysfunction in the 74 RA patients. Finally, we assessed associations between biomarkers of endothelial dysfunction and ultrasonographically determined common carotid artery intima–media thickness and plaque in RA. The three biomarkers of endothelial dysfunction, as well as hs-CRP, IL-1, IL-6 and tumor necrosis factor-α, were higher in patients than in control individuals (*P *< 0.0001). Patients were also older, exercised less and had a greater waist circumference, blood pressure and triglyceride levels (*P *≤ 0.04). Five patients had diabetes. Differences in endothelial function were no longer significant between patients and controls (*P *= 0.08) only after both traditional and nontraditional cardiovascular risk factors were controlled for. In the 74 RA patients, IL-6 predicted levels of all three biomarkers (*P *≤ 0.03), and rheumatoid factor titres and low glomerular filtration rate (GFR) both predicted levels of VCAM-1 and ICAM-1, independent of traditional cardiovascular risk factors (*P *≤ 0.02). VCAM-1 was associated with common carotid artery intima–media thickness (*P *= 0.02) and plaque (*P *= 0.04) in RA. Patients had impaired endothelial function, less favourable traditional cardiovascular risk factor profiles, and higher circulating concentrations of hs-CRP and cytokines compared with healthy control individuals. Both traditional and nontraditional cardiovascular risk factors contributed to the differences in endothelial function between RA patients and healthy control individuals. IL-6, rheumatoid factor titres and low GFR were independently predictive of endothelial dysfunction in RA. Disease-modifying agents that effectively suppress both cytokine and rheumatoid factor production, and interventions aimed at preserving renal function may attenuate cardiovascular risk in RA.

## Introduction

Cardiovascular disease is an increasingly recognized contributor to excess morbidity and mortality in rheumatoid arthritis (RA) [1-5]. Traditional cardiovascular risk factors do not adequately accunt for the extent of cardiovascular disease in RA [3,5]. Although hypertension and age are potential additional contributors to cardiovascular events in this disease [6], markers of current and cumulative inflammation (white cell counts and radiographic joint damage, respectively) are associated with ultrasonographically determined subclinical atherosclerosis [7,8] – a predictor of cardiovascular events [9].

Atherosclerosis often develops subclinically over prolonged periods of time; therefore, it may be too insensitive to show associations with recently acquired or temporarily active modifiable cardiovascular risk factors, such as systemic inflammation secondary to recent onset or uncontrolled RA. Clearly, other outcome variables that can identify patients at risk for cardiovascular disease at any point in time are needed in RA.

One such potential marker is endothelial dysfunction – an essential step in atherogenesis [10]. Most if not all risk factors that are related to cardiovascular disease are also associated with endothelial dysfunction, and the process is reversible with effective treatment of operative risk factors [10]. Endothelial status may be regarded as an integrated index of all atherogenic and atheroprotective factors present in an individual [10].

Several methods have been employed to assess endothelial function. Using ultrasonographically measured brachial artery flow mediated dilatation or vasodilatory responses to intrabrachial artery infusion of acetylcholine, some [11-14] but not all investigators [15] have shown impaired endothelial function in RA. Endothelial dysfunction was related to inflammation [12] and HLA-DR1 [11], and was found to improve with infliximab treatment [13]. An alternative method to assess endothelial function involves the measurement of biomarkers of endothelial activation and dysfunction (circulating vascular cell adhesion molecule [VCAM]-1, intercellular adhesion molecule [ICAM]-1, and endothelial leucocyte adhesion molecule [ELAM]-1 [or selectin]) [16-20]. Elevated circulating adhesion molecules are associated with cardiovascular risk factors [17] and predict atherosclerosis and cardiovascular events [18-20]. The measurement of circulating adhesion molecules may not add much predictive information to that provided by more established risk factors in the general population [21]. In contrast, it has been reported that such biomarkers play a more important role than traditional risk factors in cardiovascular disease in RA [22,23]. Important in this context is that high circulating adhesion molecule levels may not only reflect synovial inflammation but also indicate exposure of the systemic vascular endothelium to high circulating cytokine concentrations [24].

To our knowledge, the relative impact of traditional versus nontraditional cardiovascular risk factors on endothelial dysfunction as assessed using biomarkers has not been reported in RA. The present study comprises three components. First, we compared biomarkers of endothelial dysfunction in 74 RA patients and 80 healthy control individuals before and after controlling for potential explanatory variables, including both traditional and nontraditional cardiovascular risk factors. Second, we investigated in the 74 RA patients the potential role played by an extensive range of patient characteristics in endothelial dysfunction. Finally, we assessed the association between biomarkers of endothelial dysfunction and ultrasonographically determined common carotid artery (CCA) intima–media thickness (IMT) and plaque [9].

## Materials and methods

### Biomarkers of endothelial dysfunction in RA patients and healthy control individuals

Seventy-six consecutive patients who fulfilled the American College of Rheumatology criteria for RA [25] were invited to participate. Only two patients refused, and the remaining 74 were included. Patients receiving lipid-lowering and antidiabetic medications were excluded. The baseline clinical and routine laboratory characteristics of the included RA patients are reported elsewhere [26]. Eighty-three individuals with no known diseases and who were not taking any medication agreed to act as controls. These were friends, patient friends and laboratory staff. Three of them were excluded because they were found to have impaired fasting glucose (plasma glucose >5.5 mmol/l); the remaining 80 were included in the study. The study was approved by the Ethics Committees for Research on Human Subjects (Medical) of the University of the Witwatersrand and the Milpark Hospital.

We recorded traditional cardiovascular risk factors in both RA patients and control individuals using previously reported methods (Table 1) [26]. We also recorded the following nontraditional cardiovascular risk factors: circulating high-sensitivity C-reactive protein (hs-CRP), cytokines (IL-1, IL-6 and tumor necrosis factor [TNF]-α), cytokine suppressant therapy (disease-modifying antirheumatic drug [DMARD] and prednisone use) and biomarkers of endothelial dysfunction (Tables 1 and 2). Blood sampling was performed on the same day that clinical data were recorded. Fasting blood samples were taken between 08:00 and 10:00 hours. All laboratory analyses except for assessments of cytokines and biomarkers of endothelial dysfunction were performed within 2 hours of sampling. These comprised lipids, hs-CRP and other laboratory tests that were performed for the second component of the study, which was conducted in the RA patients only (see below). Blood samples drawn for determination of cytokines and biomarkers of endothelial dysfunction were stored at -70°C before laboratory testing. Cytokines and adhesion molecules were measured using enzyme-linked immunosorbent assays (Hiss Diagnostics GmbH, Freiburg, Switzerland). The intra-assay and inter-assay coefficients of variation (respectively) were 5.1% and 8.6% for IL-1, 3.4% and 5.2% for IL-6, 6.9% and 7.4% for TNF-α, 3.1% and 5.2% for VCAM-1, 4.1% and 7.7% for ICAM-1, and 5.4% and 6.0% for ELAM-1.

#### Statistical analysis

The traditional and nontraditional risk factors were compared using the Mann–Whitney U-test (continuous variables) and the χ^2 ^test (dichotomous variables; Table 1). Apart from hs-CRP, cytokines and cytokine suppressant therapy (DMARD and glucocorticoid use), several traditional cardiovascular risk factors differed between RA patients and control individuals. The ability of traditional and nontraditional cardiovascular risk factors to account for differences in the levels of biomarkers of endothelial dysfunction between RA patients and control individuals was assessed in logistic regression models (Table 2), using RA status as the dependent variable (RA = 1, non-RA control = 0). Continuous variables that were not normally distributed were logarithmically transformed. *P *< 0.05 was considered statistically significant.

### Relationship between patient characteristics and biomarkers of endothelial dysfunction in RA patients

For the second component of the study, recorded variables other than cytokines and biomarkers of endothelial dysfunction are summarized in Tables 3 and 4. A descriptive analysis of these variables in the current cohort was previously reported [26]. Although 10 patients were on thyroxine replacement therapy and eight had subclinical hypothyroidism (thyrotropin >4 μIU/ml and normal thyroxine levels) [27], none had clinical hypothyroidism (decreased thyroxine levels) at the time of the study. IgM rheumatoid factor was determined using an immunoturbidimetric test on the Olympus AU 600 analyzer. The intra-assay and inter-assay coefficients of variation for rheumatoid factor were 3.4% and 4.6%, respectively.

#### Statistical analysis

Associations between patient characteristics and biomarkers of endothelial dysfunction were first assessed by univariate analyses comprising the Spearman correlation coefficients (continuous variables; Table 3) and the Mann–Whitney U-test (dichotomous variables; Table 4). Because many univariate analyses were conducted in this component of the study, *P *< 0.01 was considered statistically significant. Because IL-6, rheumatoid factor and low glomerular filtration rate (GFR) were associated with biomarkers of endothelial dysfunction, their potential roles as predictors of endothelial dysfunction were further assessed after controlling for traditional cardiovascular risk factors in multivariable regression models (Table 5). Continuous variables that were not normally distributed were logarithmically transformed. In these multivariable models, *P *< 0.05 was considered statistically significant.

### Associations between biomarkers of endothelial dysfunction and common carotid artery intima–media thickness and plaque in RA patients

The CCAs were evaluated using high resolution B-mode ultrasound. Details of this investigation in the present cohort were previously reported [26].

#### Statistical analysis

The associations between biomarkers of endothelial dysfunction and CCA IMT and plaque were determined using the Spearman correlation coefficient and the Mann–Whitney U test, respectively. *P *< 0.05 was considered statistically significant.

## Results

### Biomarkers of endothelial dysfunction in RA patients and healthy control individuals

The recorded baseline characteristics in the RA patients and control individuals comprised traditional cardiovascular risk factors, hs-CRP and cytokines (Table 1). RA patients were younger, exercised less, had a higher waist circumference, higher systolic and diastolic blood pressures, and higher triglyceride levels than did control individuals. Five RA patients had diabetes that was being treated with dietary intervention only. With regard to nontraditional cardiovascular risk factors, hs-CRP, IL-1, IL-6 and TNF-α concentrations were higher in patients than in control individuals, and DMARD and prednisone were used by 56 and 11 RA patients, respectively. No patient was being treated or had been treated with biological agents at the time of the study.

Results for biomarkers of endothelial dysfunction in patients and control individuals are shown in Table 2. In univariate analysis, all three biomarkers of endothelial dysfunction (VCAM-1, ICAM-1 and ECAM-1) were higher in patients than in control individuals. These differences remained significant after controlling for cytokine suppressant agent (DMARD and prednisone) use (model 1) or traditional cardiovascular risk factors (model 2). After controlling for nontraditional cardiovascular risk factors, the differences in ELAM-1 between patients and control individuals were no longer significant (model 3). When traditional and nontraditional cardiovascular risk factors were simultaneously controlled for, the differences in levels of all three biomarkers of endothelial dysfunction were no longer significant between patients and control individuals (model 4).

### Relationship between patient characteristics and biomarkers of endothelial dysfunction in RA patients

In univariate analysis, VCAM-1 was related to rheumatoid factor titres and low GFR, ICAM-1 to rheumatoid factor titres and IL-6, and ELAM-1 to IL-6 (Table 4). None of the other recorded baseline characteristics in the 74 RA patients were associated with biomarkers of endothelial dysfunction (Tables 4 and 5).

After controlling for traditional cardiovascular risk factors in multivariable regression models, IL-6 was predictive of all three biomarkers of endothelial dysfunction, and rheumatoid factor titre and low GFR were both predictive of VCAM-1 and ICAM-1 (Table 5). Additional controlling for thyrotropin levels did not materially alter these models (data not shown).

### Associations between biomarkers of endothelial dysfunction and common carotid artery intima–media thickness and plaque in RA patients

As previously reported, the median (range) CCA IMT was 0.654 mm (0.496–1.150 mm), and 23 (31%) patients had plaque [26]. Twenty-one (28%) patients had a CCA IMT under 0.600 mm and no plaque. The role of clinical and routine laboratory characteristics as predictors of common carotid atherosclerosis in the present cohort was also previously reported [26]. VCAM-1 concentrations correlated with the CCA IMT (r_s _= 0.280; *P *= 0.016) and were higher in patients with plaque than in those without plaque (769 pg/ml [391–2073 pg/ml] versus 703 pg/ml [445–2001 pg/ml]; *P *= 0.043). The associations between CCA findings and other biomarkers did not achieve statistical significance.

## Discussion

### Biomarkers of endothelial dysfunction in RA patients and healthy control individuals

We found that biomarkers of endothelial dysfunction were markedly higher in RA patients than in healthy control individuals. Increased circulating adhesion molecule concentrations have been reported in RA [23,24,28,29]. Our patients also had higher hs-CRP and IL-1, IL-6 and TNF-α concentrations than did control individuals, but these nontraditional cardiovascular risk factors did not fully account for the differences in biomarkers of endothelial dysfunction between patients and control individuals. Indeed, the RA patients also had generally less favourable traditional cardiovascular risk factor profiles than healthy control individuals.

The younger age of the healthy control individuals included reflects the difficulties in recruiting healthy aged persons who are not taking any medication. Hence, we controlled for age as well as other traditional cardiovascular risk factors when assessing the differences in biomarkers of endothelial dysfunction between patients and control individuals. Of interest, the body mass indices were similar in both groups, but patients had higher waist circumference, blood pressure and triglyceride levels. The latter are features of the metabolic syndrome [5,30]. Although differences in age and exercise habits might have contributed to these findings, features of the metabolic syndrome also cluster in RA because of inflammation-induced insulin resistance [5,30].

In multivariable models, traditional cardiovascular risk factors attenuated the differences in biomarkers of endothelial dysfunction between patients and control individuals to a lesser extent than did nontraditional cardiovascular risk factors. However, the differences in endothelial function between patients and control individuals were no longer significant only after both traditional and nontraditional cardiovascular risk factors had been controlled for. Our results also show the need for comprehensive assessment of cardiovascular risk factors in healthy individuals when comparing their endothelial function with that in RA patients.

### Relationship between patient characteristics and biomarkers of endothelial dysfunction in RA patients

We investigated the potential role of an extensive range of patient characteristics in endothelial dysfunction in RA. IL-6, rheumatoid factor titre and low GFR predicted endothelial dysfunction, as assessed using biomarkers.

In multivariable analysis, IL-6 predicted endothelial dysfunction independent of traditional cardiovascular risk factors. Chronic cytokine release from inflamed joints was previously implicated in the increased production of adhesion molecules by endothelial cells in RA [4,31]. Circulating cytokines could impair endothelial function directly [4] or through their effects on insulin sensitivity and on CRP and fibrinogen (a major determinant of erythrocyte sedimentation rate [32]) production by the liver [4]. In the present cohort of unselected RA patients, IL-6 was more strongly associated with endothelial dysfunction than were CRP, erythrocyte sedimentation rate and insulin resistance. In contrast to IL-6, circulating IL-1 and TNF-α concentrations were not associated with endothelial dysfunction. IL-1 and TNF-α are major proinflammatory cytokines in RA joints and stimulate IL-6 production by synovial fibroblasts [33-37], whereas IL-6 is a major circulating cytokine in RA that induces the acute phase response, production of immunoglobulins by B cells and neuroendocrine alterations [33,34,37,38]. IL-6 promotes adhesion molecule expression and stimulates macrophages to secrete monocyte chemotactic protein-1 [39]. Circulating IL-6 concentrations also predict cardiovascular disease in the general population, independent of hs-CRP levels [39].

Apart from IL-6, rheumatoid factor was also predictive of endothelial dysfunction independent of traditional cardiovascular risk factors in the present cohort. The mechanism underlying the strong association between rheumatoid factor and endothelial dysfunction in RA cannot be discerned from our data. However, rheumatoid factor may directly cause endothelial injury [31]. Direct evidence for a role for humoral immunity in atherosclerosis was found by George and coworkers [40]. In their study repeated intraperitoneal administration of IgG from serum of mice immunized with heat shock protein 65 enhanced fatty streak formation in mice in comparison with their control anti-bovine serum albumin injected littermates [40]. Rheumatoid factor is produced by B cells that are highly effective at presenting antigens to T cells [41] and T-cell activation in rheumatoid synovium is B-cell dependent [42]. The recently reported remarkable efficacy of B-cell depletion in rheumatoid factor positive RA with rituximab [43] indicates that B cells are key contributors to the immunopathogenesis of RA. In a recent autopsy report on two RA patients with coronary artery disease the coronary plaques and adventitia contained large numbers of B cells, whereas in coronary artery disease it is typical for lymphocytic infiltrates to consist almost exclusively of T cells [44]. These reports and our findings support a role for humoral mechanisms in RA atherogenesis.

Finally, a low GFR also predicted endothelial dysfunction in our RA patients. Although only four (5%) patients had an elevated serum creatinine (>115 μmol/l), GFR estimation using the Cockcroft–Gault equation [45] revealed that 16 (22%) patients had a GFR under 60 ml/min [26], which is indicative of chronic kidney disease. The high prevalence of cardiovascular disease in individuals with chronic kidney disease has been amply reported [45]. This is attributable to the high prevalence of traditional risk factors such as older age, high total cholesterol, low high-density lipoprotein cholesterol and diabetes, as well as to nontraditional risk factors such as oxidant stress, inflammation and, to a lesser extent, hyperhomocysteinaemia. In the present cohort, varimax rotated factor analysis confirmed strong relationships between low GFR, age and hyperhomocysteinaemia [26]. Also, independent of age as well as other traditional cardiovascular risk factors, a low GFR remained independently predictive of endothelial dysfunction in our patients whereas hs-CRP was not associated with circulating adhesion molecules. In support of an important role of oxidant stress in cardiovascular disease complicating chronic kidney disease, both vitamin E 800 U daily and acetylcysteine 600 mg twice daily were shown to decrease cardiovascular events in randomized controlled trials in haemodialysis patients [45]. Whether such interventions could decrease cardiovascular disease in RA may deserve further study.

### Associations between biomarkers of endothelial dysfunction and common carotid artery intima–media thickness and plaque in RA patients

In a previous study conducted by Wallberg-Jonsson and coworkes [23] in 39 RA patients, ICAM-1 and selectin concentrations were found to be related to ultrasonographically detected CCA and femoral artery plaque as well as to haemostatic factors of endothelial origin. We found that VCAM-1 was associated with ultrasonographically determined CCA IMT and plaque. Taken together, these data further support the contention that circulating adhesion molecules are linked to cardiovascular disease in RA.

### Study limitations

We assessed cardiovascular risk comprehensively and our results are in keeping with previously reported paradigms of cardiovascular disease in RA [4,5,31]. However, our findings must be reproduced in a longitudinal study and investigations of a larger cohort is likely to reveal more independent associations between biomarkers of endothelial dysfunction and cardiovascular risk factors.

## Conclusion

We found that the biomarkers of endothelial dysfunction VCAM-1, ICAM-1 and ELAM-1 were higher in 74 RA patients than in 80 healthy control individuals. In multivariable regression models these differences could be accounted for by nontraditional cardiovascular risk factors (high hs-CRP, IL-1, IL-6 and TNF-α) and unfavourable traditional cardiovascular risk factor profiles in RA patients. IL-6, rheumatoid factor titre and low GFR predicted endothelial dysfunction, as assessed by biomarkers, independent of traditional cardiovascular risk factor in the 74 RA patients. VCAM-1 was associated with CCA atherosclerosis in RA. Those DMARDs that are most effective at suppressing both cytokine and rheumatoid factor production may also be most effective in protecting against cardiovascular disease in RA. In addition, interventions aimed at preserving renal function may need to be considered in cardiovascular disease prevention in RA.

## Abbreviations

CCA = common carotid artery; DMARD = disease-modifying antirheumatic drug; ELAM = endothelial leucocyte adhesion molecule; GFR = glomerular filtration rate; hs-CRP = high-sensitivity C-reactive protein; ICAM = intercellular adhesion molecule; IL = interleukin; IMT = intima–media thickness; RA = rheumatoid arthritis; TNF = tumour necrosis factor; VCAM = vascular cell adhesion molecule.

## Competing interests

The author(s) declare that they have no competing interests.

## Authors' contributions

PD conceived the study, collected the data, performed the statistical analysis and drafted the manuscript. BJ participated in the study design, interpretation of the data and drafting the manuscript. SS conducted the immunoassays.

## Figures and Tables

**Table 1:** Cardiovascular risk factors in RA patients and control individuals

Risk factors	Controls	Patients	*P*
Traditional risk factors			
Age (years)	44 (20–87)	57 (27–81)	<0.0001
Women (*n *[%])	64 (80)	64 (86)	0.3
Caucasian:asian (*n*:*n*)	87: 6	71: 9	0.3
Smokers (*n *[%])	25 (31)	17 (23)	0.3
Smoking (cigarettes/day)	0 (0–50)	0 (0–40)	0.5
Alcohol users (*n *[%])	37 (47)	26 (35)	0.2
Alcohol (units/week)	0 (0–40)	0 (0–35)	0.4
Exercisers (*n *[%])	43 (54)	19 (26)	0.0004
Exercise (hours/week)	1 (0–20)	0 (0–7)	0.0002
Diabetes (*n *[%])	0 (0)	5 (7)	0.02
Body mass index (kg/m^2^)	24.3 (17.9–33.8)	23.7 (17.9–38.3)	1.0
Waist (cm)	81 (60–109)	85 (66–120)	0.01
SBP (mmHg)	115 (83–150)	123 (98–164)	<0.0001
DBP (mmHg)	71 (47–100)	82 (67–109)	<0.0001
Total cholesterol (mmol/l)	5.3 (2.1–7.9)	5.1 (3.5–7.5)	0.4
HDL-cholesterol (mmol/l)	1.5 (0.4–2.3)	1.6 (0.8–2.6)	0.4
Triglycerides (mmol/l)	1.0 (0.5–4.7)	1.2 (0.5–2.6)	0.04
Nontraditional risk factors			
hs-CRP (mg/l)	1.6 (0.3–8.9)	10.8 (0.3–256)	<0.0001
IL-1 (pg/ml)	0.7 (0–42.7)	3.5 (0.1–323)	<0.0001
IL-6 (pg/ml)	0.8 (0.1–8.2)	5.4 (0.5–186.3)	<0.0001
TNF-α (pg/ml)	0.2 (0.2–32.6)	3.0 (0.3–93.2)	<0.0001
Cytokine suppressant therapy			
DMARD use (*n *[%])	0 (0)	56 (76)	<0.0001
Prednisone use (*n *[%])	0 (0)	11 (15)	0.0003

Results are expressed as median (range) unless indicated otherwise. Data were analyzed using the Mann–Whitney U-test (continuous variables) or the χ^2 ^test (dichotomous variables). DBP, diastolic blood pressure; DMARD, disease-modifying antirheumatic drug; HDL, high-density lipoprotein; hs-CRP, high-sensitivity C-reactive protein; IL, interleukin; RA, rheumatoid arthritis; SBP, systolic blood pressure; TNF, tumour necrosis factor.

**Table 2:** Biomarkers of endothelial dysfunction in rheumatoid arthritis patients and control individuals

Marker	Controls	Patients	Unadjusted	*P *(multivariable adjusted)			
				
				Model 1^a^	Model 2^b^	Model 3^c^	Model 4^d^
VCAM-1 (pg/ml)	506 (253–1067)	747 (391–2077)	<0.0001	<0.0001	<0.0001	<0.0001	0.08
ICAM-1 (pg/ml)	231 (82–857)	366 (135–993)	<0.0001	0.0002	<0.0001	0.0005	0.08
ELAM-1 (pg/ml)	48 (7–178)	58 (12–149)	<0.0001	0.02	0.02	0.7	0.08

Results are expressed as median (range). Unadjusted comparisons were done using the Mann–Whitney U-test and adjustments for potentially explanatory variables were made using logistic regression models. ^a^Adjusted for disease-modifying antirheumatic drug and prednisone use. ^b^Adjusted for traditional risk factors (age; sex; race; smoking, alcohol and exercising status; diabetes; waist; systolic blood pressure; total cholesterol; high-density lipoprotein cholesterol; triglycerides). ^c^Adjusted for nontraditional risk factors (high sensitivity C-reactive protein, interleukin IL-1, IL-6 and tumour necrosis factor-α). ^d^Adjusted for traditional and nontraditional risk factors. ELAM, endothelial leukocyte adhesion molecule; ICAM, intercellular adhesion molecule; VCAM, vascular adhesion molecule.

**Table 3:** Spearman correlations among biomarkers and potential cardiovascular risk factors

Cardiovascular risk factor	VCAM-1 (pg/ml)	ICAM-1 (pg/ml)	ELAM-1 (pg/ml)
Demographic			
Age (years)	0.279	0.149	0.148
Lifestyle			
Smoking (cigarettes/day)	0.209	0.253	0.062
Alcohol (units/week)	0.209	0.112	-0.072
Exercise (hours/week)	0.034	0.085	-0.055
Systemic inflammation			
hs-CRP (mg/l)	0.289	0.189	0.217
ESR (mm/hour)	0.217	0.175	0.195
Disease duration (years)	0.138	0.069	0.079
Disease severity			
Radiographic score	0.157	-0.012	0.092
Rheumatoid factor (IU/ml)	0.319**	0.363**	0.290
Cytokines			
TNF-α (pg/ml)	0.107	0.204	0.019
IL-1 (pg/ml)	-0.123	-0.030	-0.120
IL-6 (pg/ml)	0.248	0.298*	0.359**
Drug therapy			
Current prednisone dose (mg/day)	0.156	0.152	0.279
Cumulative prednisone dose (mg)	0.042	0.008	0.071
Cumulative pulsed MP (mg)	-0.051	-0.155	-0.096
Metabolic syndrome			
Waist circumference (cm)	0.036	0.249	0.127
SBP (mmHg)	0.159	0.011	0.019
DBP (mmHg)	-0.042	-0.073	-0.097
HDL-cholesterol (mmol/l)	-0.093	-0.103	-0.091
Triglycerides (mmol/l)	0.186	0.181	0.079
QUICKI	-0.088	-0.035	-0.019
Uric acid (mmol/l)	0.208	0.271	0.260
Others			
Haemoglobin (g/dl)	-0.139	-0.130	-0.093
Leucocytes (× 10^6^/l)	-0.017	-0.096	0.199
Polymorphonuclear cells (×10^6^/l)	0.041	-0.092	0.212
Platelets (10^9^/l)	-0.025	-0.142	0.104
Homocysteine (μmol/l)	0.124	0.236	0.277
Thyrotropin (μIU/ml)	-0.132	-0.007	0.020
LDL-cholesterol (mmol/l)	-0.139	-0.078	0.175
Apolipoprotein(a) (mg/l)	0.097	0.004	0.063
GFR (ml/min)	-0.285*	-0.144	-0.121
Urinary albumin/creatinine (mg/mmol)	-0.182	-0.163	-0.031

DBP, diastolic blood pressure; ELAM, endothelial leukocyte adhesion molecule; ESR, erythrocyte sedimentation rate; GFR, glomerular filtration rate; HDL, high-density lipoprotein; hs-CRP, high-sensitivity C-reactive protein; ICAM, intercellular adhesion molecule; IL, interleukin; LDL, low-density lipoprotein; MP, methylprednisolone; QUICKI, Quantitative Insulin Sensitivity Check Index; SBP, systolic blood pressure; TNF, tumour necrosis factor; VCAM, vascular adhesion molecule. *P ≤ 0.01; **P < 0.006.

**Table 4:** Number of patients and biomarkers of endothelial dysfunction by sex, race, diabetes and medications used

	Number	VCAM-1 (pg/ml)	ICAM-1 (pg/ml)	ELAM-1 (pg/ml)
Sex				
Female	64	750 (391–2073)	356 (135–993)	58 (12–144)
Male	10	698 (463–1814)	480 (235–805)	60 (38–149)
Race				
Caucasian	68	750 (391–2073)	366 (135–993)	61 (12–149)
Asian	6	572 (463–971)	352 (235–699)	49 (35–82)
Diabetes				
Yes	5	674 (554–1338)	376 (235–455)	55 (52–129)
No	69	749 (391–2073)	365 (135–993)	59 (12–149)
COX-2 inhibitor use				
Yes	20	689 (475–2073)	371 (145–749)	79 (25–129)
No	54	750 (391–2001)	363 (135–993)	55 (12–149)
Traditional NSAID use				
Yes	19	766 (493–2001)	366 (135–993)	68 (16–149)
No	55	703 (391–2073)	365 (145–761)	55 (12–129)
Aspirin use				
Yes	6	719 (463–2073)	346 (219–595)	40 (23–94)
No	68	747 (391–2001)	366 (135–993)	62 (12–149)
Oestrogen use				
Yes	24	673 (445–2001)	311 (181–993)	53 (12–83)
No	50	786 (391–2073)	389 (135–805)	64 (20–149)
DMARD use				
Yes	56	726 (391–2073)	368 (145–805)	56 (12–149)
No	18	816 (567–2001)	358 (135–993)	64 (34–115)
Prednisone use				
Yes	11	930 (391–2073)	455 (181–761)	94 (23–129)
No	63	724 (445–2001)	361 (135–993)	55 (12–149)
Antihypertensive agent use				
Yes	23	744 (569–2073)	414 (219–805)	66 (16–149)
No	51	758 (391–2001)	350 (135–993)	54 (12–137)

Results for biomarkers are expressed as median (range). Data were analyzed using the Mann–Whitney U test. No comparisons were significant at *P *≤ 0.01. COX-2, cyclooxygenase-2; DMARD, disease-modifying antirheumatic drug; ELAM, endothelial leukocyte adhesion molecule; ICAM, intercellular adhesion molecule; NSAID, nonsteroidal anti-inflammatory drug; VCAM, vascular adhesion molecule.

**Table 5:** Partial correlation coefficients between IL-6, rheumatoid factor and glomerular filtration rate, and biomarkers of endothelial dysfunction in rheumatoid arthritis patients

Marker	IL-6		Rheumatoid factor		GFR	
	
	R^a^	*P*	R^a^	*P*	R^a^	*P*
VCAM-1 (pg/ml)	0.262	0.04	0.345	0.006	-0.299	0.02
ICAM-1 (pg/ml)	0.263	0.04	0.373	0.003	-0.351	0.005
ELAM-1 (pg/ml)	0.430	0.0005	0.222	0.09	-0.223	0.07

^a^The correlation coefficients shown are controlled for traditional risk factors (age; sex; race; smoking, alcohol and exercising status; diabetes; waist; systolic blood pressure; total cholesterol; high-density lipoprotein cholesterol; triglycerides) in multivariable regression models. ELAM, endothelial leukocyte adhesion molecule; GFR, glomerular filtration rate; ICAM, intercellular adhesion molecule; IL, interleukin; VCAM, vascular adhesion molecule.
